# Nanomaterials modulate stem cell differentiation: biological interaction and underlying mechanisms

**DOI:** 10.1186/s12951-017-0310-5

**Published:** 2017-10-25

**Authors:** Min Wei, Song Li, Weidong Le

**Affiliations:** 1 Liaoning Provincial Center for Clinical Research on Neurological Diseases, The First Affiliated Hospital, Dalian Medical University, Dalian, 116021 People’s Republic of China; 2 Liaoning Provincial Key Laboratory for Research on the Pathogenic Mechanisms of Neurological Diseases, The First Affiliated Hospital, Dalian Medical University, Dalian, 116021 People’s Republic of China; 3 Collaborative Innovation Center for Brain Science, The First Affiliated Hospital, Dalian Medical University, Dalian, 116021 People’s Republic of China

**Keywords:** Nanomaterials, Stem cells, Differentiation

## Abstract

Stem cells are unspecialized cells that have the potential for self-renewal and differentiation into more specialized cell types. The chemical and physical properties of surrounding microenvironment contribute to the growth and differentiation of stem cells and consequently play crucial roles in the regulation of stem cells’ fate. Nanomaterials hold great promise in biological and biomedical fields owing to their unique properties, such as controllable particle size, facile synthesis, large surface-to-volume ratio, tunable surface chemistry, and biocompatibility. Over the recent years, accumulating evidence has shown that nanomaterials can facilitate stem cell proliferation and differentiation, and great effort is undertaken to explore their possible modulating manners and mechanisms on stem cell differentiation. In present review, we summarize recent progress in the regulating potential of various nanomaterials on stem cell differentiation and discuss the possible cell uptake, biological interaction and underlying mechanisms.

## Background

Stem cells are primitive cells that have the potential to self-renew and develop into different specialized functional cells. According to its developmental stage, stem cells can be classified into two broad types, embryonic stem cells (ESCs) and somatic stem cells (SSCs) [1]. ESCs are derived from the inner cell mass of blastocysts [2, 3]. With similar characteristics of ESCs, induced pluripotent stem cells (iPSCs) are produced from somatic cells by genetically reprogrammed to a ESCs-like state by introducing the expression of certain genes and factors [4]. ESCs and iPSCs are pluripotent stem cells that have the greatest differentiation potential and infinite self-renewal capacity [5, 6]. SSCs, derived from adult tissues, are more accessible, but less potent than ESCs and iPSCs [7]. In recent years, with the continuous research of stem cells, more and more types of SSCs can be isolated from bone marrow, adipose tissues, cord blood and neural tissues [8–11]. Mesenchymal stem cells (MSCs) and adipose-derived stem cells (ADSCs) as well as neural stem cells (NSCs) have become attractive stem cell source for tissue regeneration and engineering without considering the ethical issues of ESCs [12].

The clinical application of stem cells, especially in cell therapy and tissue engineering, depends on the regulation and control of cell differentiation into specific cell types [13]. In the past decade, great efforts have been made to manipulate the differentiation of stem cells into numerous types of cells, such as osteoblast cells, neurocytes, adipocytes and cardiomyocytes [14–16]. However, the low differentiation efficiency and success rate limits the development of stem cell differentiation for stem cell therapy. Additionally, undifferentiated ESCs after implantation in vivo increase the risk of teratoma, so it is important to allow committed differentiation of ESCs into specific lineages prior implantation for a safe use in cell-based therapies [17, 18]. Thus, there is an urgent need to develop strategies to improve the efficiency of directed differentiation of stem cells into specified cell types.

Nanomaterials are materials with a microstructure the characteristic length scale (at least one dimension) of which is within the nanometer range (~ 1–100 nm). Nanomaterials have been widely used to manipulate the cell behavior due to their small size, ease of synthesis and versatility in surface functionalization [19–21]. During the last decade, various nanomaterials, including liposomes [22], quantum dots [23, 24], carbon nanotubes (CNTs) [25], graphene (GR) [26], silica nanoparticles [27], titanium dioxide nanoparticles (TiO_2_) [28], silver nanoparticles (AgNPs) [29], gold nanoparticles (AuNPs) [30], iron oxide nanoparticles (IONPs) [31], DNA nanostructures [32], have been intensively explored in both biological and medical fields.

The rapid development of nanotechnology provides a great prospect for the development of novel nanomaterials with modulating potential on stem cell differentiation. In fact, various types of nanomaterials have been identified to regulate the differentiation of stem cells (i.e. ESCs, iPSCs and MSCs) into different types of cells, including adipocytes, cardiomyocytes, osteoblast cells, and neural cells through different mechanisms [33–37]. The extracellular microenvironment is considered to play an important role in influencing the function and fate of stem cells [11]. Engineered nanomaterials can mimic the stem cell environment and modulate stem cell differentiation [38]. The suppletion of specific differentiation factors such as growth factors and bioactive molecules into the medium is the widely accepted route to promote stem cell differentiation [39]. Recently, accumulating evidence has indicated that some nanomaterials, such as functionalized CNTs and GR, can facilitate stem cell proliferation and differentiation even without the need of specific media containing extra supplements [40, 41]. Furthermore, nanomaterials with surface chemical modifications can also modulate the specific properties of stem cells for differentiation. In this review, we summarize recent research progress in the modulating effects of nanomaterials on stem cell differentiation and discuss the possible modulating manners and underlying mechanisms.

### Nanomaterials-modulated stem cell differentiation

#### Metal nanoparticles

##### AuNPs

Due to their intrinsic properties such as well-controlled size and surface- functionalization, AuNPs have been widely used in biomedical fields for drug/gene delivery, biosensors, imaging, and photothermal therapy [42, 43]. The internalized AuNPs (with different surface modification or payload) may interact with proteins located in the cytoplasm, or serve as mechanical stimuli that trigger a series of biological alterations and modulate cell behaviors [34, 44, 45]. The cellular effects of AuNPs on the differentiation of stem cells have been investigated; various forms of AuNPs (sizes in 20–70 nm, surface modified with citrate, chitosan or fibronectin, etc.) have been reported to modulate the differentiation of stem cells (ADSCs, MSCs, ESCs, MSCs) into osteoblasts or cardiomyocytes [34, 46–49].

Increasing evidence suggests that AuNPs possess an inherent ability to promote the differentiation of stem cells. The size, shape and surface modifications of AuNPs can impact the cellular uptake of particles into stem cells, and consequently influence their modulating effects on stem cell differentiation. For instance, 30 and 50 nm sphere AuNPs have been proved to be most efficient among all sizes on osteogenic differentiation of hADSCs, while 40 and 70 nm sphere AuNPs, 70 nm Au nanorods coated with bovine serum albumin (BSA) affect the osteogenic differentiation of hMSCs obviously [44, 47]. In addition to the intrinsic properties of AuNPs themselves, charge and specific chemical moieties on nanomaterials surface may also contribute remarkably towards direction of stem cell fate [50]. Nanomaterials with modified surfaces can also be chemically altered to improve specific properties for enhanced cell–matrix interactions. For example, chitosan-conjugated AuNPs can promote the osteogenic differentiation of human ADSCs (hADSCs) through the Wnt/β-catenin signaling pathway [49]. Fibronectin-coated AuNPs as adhesion sites deliver electrical stimulation on the differentiation of human ESCs (hESCs) in vitro and direct induce osteogenic differentiation [48]. Additionally, AuNPs can also be utilized for cardiac differentiation. AuNPs-loaded functionalized nanofibers scaffold can couple adequate electrical, mechanical, biological or chemical properties, leading to enhanced cardiomyogenic differentiation of hMSCs [46, 51]. As for the underlying molecular mechanisms, further study has shown that AuNPs promote osteogenic differentiation of mouse MSCs (mMSCs) through p38 mitogen-activated protein kinase (MAPK) pathway [34]. Notably, although AuNPs with different sizes have been tried for stem cell differentiation, their side effects should not be neglected due to their non-biodegradable nature [52]. AuNPs modified onto three-dimension (3D) scaffolds to deliver electrical stimulation for specific stem cell differentiation seems to be a more reliable method.

##### AgNPs

Due to its remarkable antibacterial activity, AgNPs have been widely used and have become one of the fastest growing nanomaterials in the biomedical fields of recent years [53]. Similar to AuNPs, AgNPs also face the challenge that they are non-biodegradable materials and cannot be degraded in cells. The role of AgNPs in the differentiation of stem cells is controversial. AgNPs (10 or 20 nm in size) show no influence on the differentiation of hADSCs and caused minimal toxicity at antimicrobial concentrations [54]. In contrast, 30 nm AgNPs do not influence the osteogenic differentiation of hMSCs, but cause certain cytotoxicity [55]. Furthermore, AgNPs has been reported to inhibit stem cell differentiation, both AgNPs with a size of 80 nm (hydro-dynamic diameter) and silver ions could attenuate the differentiation of hMSCs to adipogenic and osteogenic even at non-toxic concentrations [56]. In contrast, another experiment leads to the opposite conclusion that AgNPs can promote osteogenic differentiation of human urine-derived stem cells (hUSCs) at a suitable concentration, in a silver ions-independent manner [57]. In addition, it can also promote the proliferation and osteogenic differentiation of mMSCs in vitro [29]. These conflicting conclusions require us to be cautious about the application of AgNPs in stem cell differentiation. Further studies are needed to find the exact underlying mechanisms. Meanwhile, AgNPs can be fabricated as drug delivery vehicles to deliver light-activated miR-148b mimic, and these miR-148-AgNPs conjugates are readily entering into cells and resulting in differentiation of hADSCs into an osteogenic linage [58]. All these findings confirm that different sizes and concentrations of AgNPs may have different effects on stem cell differentiation; therefore suitable size and concentration as well as surface modification are critical manners in consideration of application of AgNPs in stem cell differentiation.

##### TiO_2_

In view of the good biocompatibility and highly ordered nanotube arrays structure, the ability of TiO_2_ nanoparticles to promote the stem cell differentiation have attracted much attention [35, 36]. Several studies have shown that TiO_2_ of specific size and shape may have a certain effect on stem cell differentiation. The spherical TiO_2_ nanoparticles have been reported to exert negative effects on cell viability and negatively affect the osteogenic differentiation of rat MSCs (rMSCs) in a size- and dose-dependent manner [59]. In contrast with the inhibiting effects of TiO_2_ nanoparticles on stem cell differentiation, TiO_2_ nanotubes have been documented to promote stem cell differentiation [36]. Since the diameter of TiO_2_ nanotubes can be synthesized variably, nanoscale geometry has been shown to influence cellular differentiation. Studies have been conducted to determine the optimal sizes of TiO_2_ nanomaterials for their regulation on cell differentiation [60]. For example, 15 nm has been indicated as an optimal length for TiO_2_ nanotube to modulate adhesion and differentiation of human hematopoietic stem cells (hHSCs) [61]. However, another one study conducted by Lv et al. [35] has demonstrated that compared with 50 and 100 nm in size, 70 nm is the optimal dimension for TiO_2_ to regulate osteogenic differentiation both in vitro and in vivo. The TiO_2_ induces differentiation of hADSCs via an epigenetic mechanism by modulating histone H3 at lysine 4 trimethylation. Further study on underlying molecular mechanisms between TiO_2_ nanotubes and stem cell differentiation has revealed that Na^+^/K^+^ transporting ATPases ATP1A2 (alpha 2 polypeptide), ATP1A3 (alpha 3 polypeptide) and mitogen-activated protein kinase kinase kinase 11 (MAP3K11) are involved in the 100 nm TiO_2_ nanotubes-induced osteogenic differentiation of rat bone marrow stromal cells [62]. Besides, TiO_2_ nanotube arrays covered with gelatin/chitosan multilayered coatings can be used as drug nanoreservoirs for bone morphogenetic protein 2 (BMP2) loading. The multilayer-coated TiO_2_ nanotube arrays are able to promote the osteoblastic differentiation of rMSCs for controlled BMP2 release [63]. In addition, TiO_2_ can fabricate as 3D scaffolds for stem cell differentiation. TiO_2_ 3D scaffolds coated with alginate hydrogel containing simvastatin or enamel matrix derivative can direct osteogenic differentiation of stem cells [14, 64]. These findings above suggest that the shape and size of TiO_2_ nanoparticles has a great influence on the differentiation of stem cells, and TiO_2_ nanotubes but not spherical TiO_2_ nanoparticles could serve as good biomaterials for the differentiation of stem cells. In addition, TiO_2_ is more suitable for use as two-dimensional (2D) substrates or 3D scaffolds for stem cell differentiation.

##### IONPs

Similar to other metal-based nanomaterials, IONPs are also proved suitable for promoting stem cell differentiation. IONPs have been confirmed to promote osteogenic differentiation of human bone-derived mesenchymal stem cells (hBMSCs) in vitro by activating MAPK signal pathway [65]. Meanwhile, another study has reported that the field-induced assemblies of magnetic γ-Fe_2_O_3_ nanoparticles can promote the differentiation of primary mouse bone marrow cells into osteoblasts [66]. The advantage of this approach is that the promoted differentiation effect is mediated by interface effect rather than internalization. Furthermore, IONPs coated with human serum albumin (HSA) can be used as non-toxic and superparamagnetic delivery for drug controlled release. Conjugated fibroblast growth factor 2 (FGF2) core–shell NIR fluorescent iron oxide/HSA magnetic nanoparticles are also effective in enhancing the proliferation of hBMSCs and promoting their differentiation toward neuronal, adipogenic or osteogenic lineages in vitro [67].

##### Other metal-based nanomaterials

Barium titanate nanoparticles with glycol-chitosan coating have a good biocompatibility to promote the ostogenic differentiation of rMSCs in the presence of the appropriate differentiation factors (osteogenic differentiation basal medium supplemented with dexamethasone, GA-1000 (gentamicin, amphotericin-B), l-glutamine, ascorbate, FBS and glycerophosphate) [68]. Additionally, metal-based composite nanomaterials have been tested for stem cell differentiation. Magnetic core-shell structures with a ZnFe_2_O_4_ core surrounded by a gold outer shell have been successfully utilized to deliver specific siRNA/pDNA to selectively direct the differentiation of NSCs into neurons or oligodendrocytes [69].

#### Carbon nanomaterials

Carbon nanomaterials, including CNTs, GR and carbon 60 (C60), are novel materials which have been widely studied and applied [70]. These new carbon materials possess several excellent physical and chemical properties, and have been applications in biological sensors, gene and drug delivery and stem cell tracking [71, 72]. In order to meet the needs of the strong demand for the discovery and development of stem cell differentiation modulator, many carbon nanomaterials have been evaluated and showed significant modulating effects on stem cell differentiation.

##### GR and graphene oxide (GO)

GR is a novel 2D carbon nanosheet with unique physical, chemical and mechanical properties that are widely used in biomedicine field [72]. GR and its derivative, GO, have recently attracted increasing interests for biology applications. GO is an oxidative derivative of GR. Epoxide, hydroxyl, carbonyl and carboxyl groups are on the basal planes and edges of GO sheet, providing strong bonding sites and bioactivities [73, 74]. GR and GO are demonstrated to be the ideal biocompatible and mechanical platforms mediating stem cells growth and differentiation [16]. Remarkably, the different surface properties of GO and GR exhibit distinct characteristics for modulating mouse iPSCs proliferation and spontaneous differentiation. GO accelerates the iPSCs differentiation, whereas GR favorably maintains the cells in an undifferentiated state [75].

GO and GR have been reported to promote the differentiation of stem cells to neurons. GO can effectively promote the differentiation of mESCs to dopamine neuron after induction of stromal cell-derived inducing activity (SDIA) [33], while GR can be used as a cell-adhesion layer for long-term differentiation of hNSCs toward neurons [76]. Meanwhile, 3D rGO-collagen hybrid scaffold is good for the enhancement of the neural differentiation of rBMSCs [77].

Moreover, owing to their ultra lightweight, tremendous strength and stability, GR and GO also have been emerged as promising nanomaterials for tissue engineering. GO and GR sheets have the potential to support and accelerate stem cell adhesion, proliferation and differentiation, such as facilitate hMSCs differentiation towards osteogenic lineage [16]. Consistently, GR provides a promising biocompatible scaffold that does not hamper the proliferation of hMSCs and accelerates their specific differentiation into bone cells even in the absence of commonly used additional growth factors such as BMP-2 [41]. Furthermore, GO-doped poly(lactic-co-glycolic acid) (PLGA) nanofiber designed as 3D scaffolds effectively promote the differentiation of hMSCs toward osteoblasts. The incorporated GO can enhance the hydrophilicity and protein-/inducer adsorption ability of the nanofibers. It not only accelerates the attachment and proliferation of hMSCs, but also induces the osteogenic differentiation [78].

Additionally, as a new type of carbon-based quantum dots, graphene quantum dots (GQD) also exert no significant influences on self-renewal potential and enhance the differentiation of rBMSCs into osteoblasts and adipocytes [79].

##### CNTs

CNTs have emerged as one of the most promising nanomaterials due to their tremendous strength, ultralight weight and high stability. CNTs can be considered as a layer of rolled GR sheet [80]. According to the numbers of GR layers, they are normally categorized as single-walled (SWNTs) or multi-walled nanotubes (MWNTs) [81]. Both SWNTs and MWNTs have no adverse effects on biocompatibility, proliferation, or differentiation of hMSC for future approaches to tissue repair/regeneration [82]. However, carboxylated-CNTs have been demonstrated to inhibit the proliferation, osteogenic/adipogenic differentiation of mMSCs in a suspended CNTs condition [83]. These conflicting results may indicate a negative impact of surface modification on CNTs-modulated differentiation of stem cells.

CNTs are good matrix materials for stem cell differentiation. Polyethylene glycol (PEG)-conjugated MWNTs layers show no cytotoxicity [40, 84, 85], and accelerate the differentiation of stem cells even without any additional differentiating factors [40]. In another study, carboxylated MWCNT-coated substrates have been reported to provide a suitable environment for the spontaneous long-term neural differentiation of hBMSCs with low cytotoxicity [86]. Also, SWCNTs films are excellent 2D nanomaterials that can enhance the cell growth and differentiation of rMSCs as a culture substrate; the variation of thickness, roughness, surface property of SWCNTs films will positively affect the growth and differentiation characteristics of MSCs, and specific cells differentiated from MSCs can be precisely controlled by altering the property of SWCNTs films [87]. Incorporated carbon nanomaterials (a mixture of GR and SWCNTs) into electrospun polycaprolactone (PCL) scaffolds can greatly improve the mechanical strength properties of the scaffolds and enhance hBMSCs growth and chondrogenic differentiation [88].

CNTs can also be designed and fabricated as novel 3D nanostructured scaffolds for stem cell proliferation and differentiation. Hydrogen treated CNTs poly(l-lactic acid) scaffolds with poly-l-lysine (PLL) coating can induce the differentiation of hBMSCs into chondrogenic more than control groups [89]. Furthermore, poly(ε-caprolactone)-CNTs composite scaffolds possess the ability to promote cardiac differentiation of hMSCs in 3D culture [90]. The incorporated CNTs can greatly improve the strength of composite scaffolds, and possess an inherent ability to promote stem cell differentiation without adverse effects on cellular activity. Despite the advantage for stem cell differentiation mentioned above, CNTs have some limitations. Pristine CNTs are insoluble and cannot be used directly; moreover, nanotubes also have some toxic effects [91]. Proper surface modification of CNTs can increase solubility and reduce toxicity, which should be taken into account when CNTs are used for stem cell differentiation.

#### Semiconductor nanomaterials

As an important semiconductor nanomaterial, silica nanoparticles can serve as a vehicle for drug delivery or gene therapy [27, 92]. It seems that silica nanoparticles lack of positive effect but even have negative effects to some extent on stem cell differentiation [93]. Previous studies have shown that the pure nanoparticles had no effect on cellular ultrastructures and adipogenic/osteogenic differentiation of rMSCs [94]. However, due to its biocompatibility, controllable particle size, tunable pore size and high loading capacity, silica nanoparticles have been explored to serve as nanocarriers to promote the stem cell differentiation [92]. Silica nanoparticles are good protein carriers and can be used as carriers for insulin delivery to induce the rMSCs differentiate into adipocytes in vitro [94]. Furthermore, the treatment of hESCs with ascorbic acid (AA)-loaded fluorescent TRITC-mesoporous silica nanoparticles results in a higher induction efficiency of stem cell differentiation and can serve as a potential tool to promote the differentiation of hESCs into cardiomyocytes [15]. Additionally, silica nanoparticles as nucleic acid carriers are also used for specific differentiation. FITC-conjugated mesoporous silica nanoparticles are fabricated as a suitable carrier to deliver hepatocyte nuclear factor 3β (HNF3β) plasmid DNA, the silica-based delivery platform can quickly induce mouse iPSCs to differentiate into hepatocyte-like cells [95]. In addition, silica also can be modified as nanocarriers to deliver pigment epithelium-derived factor (PEDF) siRNA to regulate the differentiation and self-renewal of cardiac stem cells [96]. Although silica nanoparticles itself does not have the ability to promote stem cell differentiation, it is worth mentioning that silica nanoparticles can be considered as a nanocarrier for stem cell differentiation applications due to their good bio-safety, biocompatibility, and high loading capacity.

#### Polymeric nanoparticles

Polymeric nanoparticles also play an important role in the differentiation of stem cells. Polymeric nanoparticles are capable of modifying active drugs, delaying and controlling the release of drugs, and being frequently used as drug-delivery systems [97]. Retinoic Acid (RA) can be controlled release from RA-hydrophilic polyethylenimine (PEI) complex nanoparticles in vitro by pH and induce the ES cell-derived neuronal differentiation [98]. Moreover, thermo-responsive RA-loaded poly (*N*-isopropylacrylamide)-co-acrylamide (PNIPAM-co-Am) polymeric nanoparticles (PCANs) can enhance hiPSC differentiation. RA can be intracellular released from RA-loaded PCANs for the thermo-responsive property and efficiently direct hiPSC differentiation into neuronal lineage [99]. Particularly, polyelectrolyte nanoparticles by electrostatic interaction of PEI and dextran sulfate can deliver RA into mouse sub-ventricular zone (SVZ) stem cells in vitro, the results have demonstrated that the RA-loaded nanoparticles can intracellular release RA and promote the differentiation of stem/progenitor cells [100]. Furthermore, their further study has shown that the RA-loaded polymeric nanoparticles could be in vivo control the differentiation of SVZ neural stem cells, the differentiation mechanism of RA-loaded nanoparticles is that the released RA from nanoparticles interacts with RA receptor (RAR), activate SAPK/JNK signaling pathway, and ultimately increase the expression of proneurogenic gene [101]. These findings may pave the way for the treatment of neurodegenerative diseases by using nanomaterials and make polymeric nanoparticles a useful delivery system of RA for neuronal differentiation.

Among the polymeric nanoparticles, chitosan offers certain advantages over other ones for drug delivery due to its biodegradability, biocompatibility, low immunogenicity and low toxicity [102]. Delivery of nucleic acid with chitosan to regulate osteogenic differentiation was also tested. Chitosan nanoparticle/hsa-miR-199a-5p agomir complexes can both modulate osteogenic differentiation of hMSCs in vitro and improve the regeneration of bone in vivo in a hypoxia inducible factor-1α (HIF-1α) pathway related manner [103]. It has been documented in another study that chitosan is a potential candidate as an efficient non-viral miRNA vector to regulate the osteogenic differentiation of MSCs; chitosan-based- microRNA nanoparticles can be a safe and effective carrier for antimiR-138 delivery to rMSCs with high transfection efficiency and significantly enhance the osteogenesis of rMSCs [104]. Direct delivery of miRNA into stem cells provides appropriate therapy of specific diseases, the high loading capacity and controlled drug-release ability make polymer nanoparticles become promising drug carriers for various differentiation purpose.

Furthermore, polymers are candidates of highly biocompatible scaffolds for stem cell differentiation. Novel spherical nano-hydroxyapatite/chitosan/gelatin 3D porous scaffolds can also enhance the proliferation and osteogenic differentiation of hiPSCs [105].

Although polymeric nanoparticles have the above advantages in stem cell differentiation, the cytotoxicity of most polymers themselves is still a question that cannot be ignored. Polymers with high molecular weight, such as high branched PEI exhibit high toxicity; low molecular weight display low toxicity yet transfection efficiency is low as well [106, 107]. Other polymeric nanoparticles like PLL, poly(diallyl-dimethyl-ammonium chloride) show similar toxicity to PEI [108]. However, chitosan and its derivatives display better biocompatibility and relatively good transfection efficiency [109, 110]. No one system meets all needs; among these polymeric nanoparticles, chitosan seems to have a better biocompatibility, which is valuable to study more.

#### DNA nanostructures

DNA nanostructures with well defined structures and uniform sizes have emerged as novel nanomaterials for biomedical applications [32]. Variety of artificial DNA nanostructures, including DNA origami, DNA tetrahedron, DNA nanotube, have been fabricated through appropriate design of DNA sequences [111–113]. DNA nanostructures show merits in low immunogenicity, good biocompatibility, controllable surface modification, reproducibility and low cost. Numerous studies have reported the potential application of DNA nanostructures for disease diagnosis and treatment, especially in the areas of biosensing and drug delivery [32, 111]. DNA nanostructures as artificial scaffolds to control the cell behavior are also tested. Assembled DNA nanotubes by self-assembly and covalently functionalized with the bioactive cell adhesion peptide Arg-Gly-Asp-Ser (RGDS) can used as artificial substrate for guiding the differentiation of mNSCs [114]. Remarkably, both nanotube architecture and peptide bioactivity synergistically promotes mNSCs differentiate into neurons rather than astrocytes [114].

### The interactions between nanomaterials and stem cells and possible underlying mechanisms

**Fig. 1:**
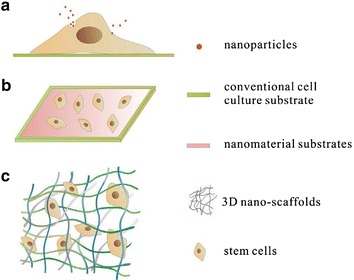
Sketch map of nanomaterials modulate the differentiation of stem cells in three ways. Nanomaterials could be used as supplements (**a**), 2D matrix (**b**) or 3D nano-scaffolds (**c**) that induce differentiation of stem cells

Up to now, the effect of nanomaterials on stem cell differentiation has been studied to a large extent by introducing stem cells into artificial microenvironment, and the application of nanomaterials in stem cell differentiation is mainly through the following ways: cell culturing with nanoparticles suspension, 2D cell culture on the surface of nanomaterials, or cell seeding and culturing on 3D nano-scaffolds (Fig. 1).

#### Nanoparticles as supplements for stem cell differentiation

**Fig. 2:**
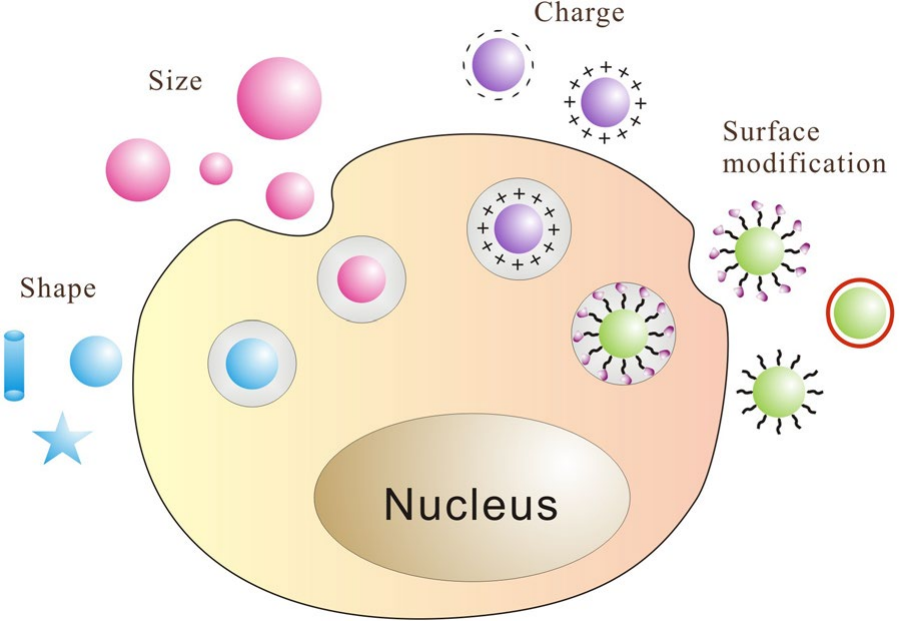
The physicochemical features of nanomaterials influence cellular uptake and consequently impact their modulating potential on stem cells differentiation

Some nanoparticles possess an inherent ability to facilitate stem cell differentiation due to their unique biological and mechanical properties. Up to now, several promising nanoparticles including AuNPs, AgNPs, GO, CNTs and silica nanoparticles have been demonstrated to promote stem cell differentiation [29, 33, 34, 86, 115]. Nanoparticles can easily transfer across cells membranes and locate in the cytoplasm, thus affecting certain cellular signaling pathways for inducing differentiation [116, 117]. The cellular pathways may differ depending on the type of nanomaterials, surface ligands and cell types. The physicochemical features of nanomaterials have a great influence on the mechanism of differentiation (Fig. 2).

##### Size and shape

Nanoparticles can serve as mechanical stimuli to activate certain signaling pathways in stem cells and thus induce differentiation. The optimal nanoparticles size for stem cell differentiation ranges between 20 and 70 nm, which probably due to the size-dependent cellular uptake rates [35, 44, 47]. Nanoparticles around 50 nm in size showed higher amounts internalized by cells [118]; smaller nanoparticles are more cytotoxic, while larger nanoparticles are less efficiently incorporated by cells [119, 120]. Furthermore, the shape of nanoparticles affects the uptake of nanoparticles by cells that may influence the stem cell differentiation [44]. The uptake rate of nanospheres is much higher than that of nanorods when their size is approximate [120]. All in all, nanoparticles were taken up by cells in a size and shape-dependent manner, thus further affecting differentiation; nanoparticles would cause mechanical signals on cells and affect stem cell differentiation due to the varying size and shape.

##### Charge and moiety

Charge and specific chemical moieties are also important for the nanomaterials to direct stem cells differentiation. Functional chemical moieties, such as amines (–NH_2_), hydroxyl (–OH) and carboxyl (–COOH) are widely present in biomolecules such as proteins, nucleic acids, lipids and polysaccharides, are important factors that affect the behavior and differentiation of stem cells [121]. For example, COOH–AuNPs treatment inhibits osteogenic differentiation, whereas those –NH_2_ and –OH groups functionalized AuNPs fail to do so [50]. In addition, different surface charges and groups affect the uptake of nanoparticles, and positively charged nanoparticles exhibit higher cell uptake and higher cytotoxicity [120]. It is noteworthy that most physical and chemical parameters are interconnected, the influence of charge may be related to size-dependent uptake, and additional surface coatings add complexity.

##### Surface modification

Different surface coating of nanomaterials can lead to different cell signaling cascades. For example, AuNPs promote osteogenic differentiation of MSCs through the p38 MAPK pathway, while chitosan-conjugated AuNPs activate the Wnt/β-catenin signaling pathway in hADSCs [34, 49]. Nanomaterials with specific surface modifications can more closely mimic the microenvironment and interaction with biological molecules and stem cells [50]. Furthermore, nanomaterials can absorb serum proteins or even bioactive molecules such as cytokines, growth factors in the physiological environment, which can promote stem cell differentiation [4, 122, 123]. In addition, surface charge and the size of nanomaterials will affect the adsorption of differentiation factors, which is due to the influence of different electrostatic interaction and area-to-volume ratio [124].

The interactions of nanoparticles and stem cells have not clearly explained, as most physicochemical parameters are entangled. It remains to further investigate the underlying mechanism of the differences relatively to the internalized nanoparticles for the differentiation of stem cells. It is worth noting that, although these nanomaterials appear to be non-toxic to cells and can promote stem cell differentiation into specific lineages, the long-term biological safety still requires further evaluation due to most of the nanomaterials cannot be degraded after cellular uptake into cells.

#### As nanocarriers for drug delivery for stem cell differentiation

Nanomaterials have shown great potential as intracellular nanocarriers for drug and nucleic acid delivery in the differentiation of stem cells. Some of the drug/chemicals have poor solubility, short half-life and/or poor penetration into cells, furthermore, naked nucleic acids cannot successfully enter cells which require the assistance of a suitable vector [125, 126]. Once inside in the cells, exogenous nucleic acids or biomacromolecules can be quickly degraded by intracellular enzymes, and small-molecule drugs rapidly metabolized by cells [125]. Nanoparticles are ideal carriers for nucleic acids/drug delivery both in vitro and in vivo [127]. Nanoparticles have the advantages of good biocompatible and ease of functionalization that they can target stem cells and release their payloads in the cytoplasm [127]. This unique feature enables nanoparticles to be used as excellent carriers to deliver drugs, nucleic acids, growth factors and other biomolecules within cells for stem cell differentiation [100, 128, 129]. Chitosan is biodegradable nanocarrier to deliver miRNA to regulate the osteogenic differentiation of MSCs, and various polymeric nanoparticles are used to deliver RA [103, 130]. In addition, inorganic nanoparticles such as AuNPs, AgNPs and silica nanoparticles are often used for drug delivery because of their load capacity, although their application is limited due to their non biodegradability [52]. For example, AgNPs is designed as a carrier to deliver miR-148b and mesoporous silica nanoparticles is used for delivery of AA [15, 58]. Nanoparticles can serve as a platform to carry different bioactive payloads with almost no influence on cell activity but a great impact on differentiation. Thus it can be seen that when drug-loaded nanomaterials enter into cells and release their payloads within cytoplasm, the payloads subsequently activate the corresponding signaling cascade. The mechanism of differentiation is mainly determined by their surface payloads of nanomaterials. Remarkably, biocompatible and biodegradable nanoparticles with the ability to target stem cells and release their payloads in the cytoplasm, and then activate signaling cascades, may be the focus of future research [117].

#### Nanomaterials as 2D matrix support for stem cells growth and differentiation

2D cell culture is a traditional method of cell culture in vitro. Physical and biological factors, such as growth factors, hormones, chemical or biological molecules, and extracellular matrix, can determine the fate of stem cells of differentiation and pluripotency. Therefore, when cultured on different cell culture substrates, stem cells may have different differentiation fates. Several researchers have reported that using nanomaterials as 2D cell culture substrate could effectively promote the differentiation of stem cells into specific lineages, and the stiffness, surface chemistry, alignment and several other parameters of the cell culture substrate (matrix) may work together to influence the fate of stem cells [16, 35, 61, 131]. Chemical and biological modifications of nanomaterials can directly influence cell–matrix interactions and ultimately manipulate the signal transduction pathways in stem cells. As an example, the aligned CNTs exhibit an enhanced proliferation and osteogenic differentiation of hMSCs probably due to the ordered nanomaterials may better mimic the orderly pattern of natural ECM [131]. Overall, nanomaterials as 2D matrix with certain geometric properties have shown positive effects on the differentiation of stem cells, which make nanomaterials good candidates for stem cell differentiation in regenerative medicine.

#### Nanomaterials as 3D nano-scaffolds for stem cell differentiation

Although classical 2D cell cultures on flat surfaces of nanomaterials can manipulate the fates of stem cells, cells proliferation and differentiation inside the body are within complex 3D microenvironments. More and more current research on the differentiation of stem cells by nanomaterials are mainly focus on 3D environments, the nanomaterials-based 3D nano-scaffolds can simulate the natural environment and usually serve the purposes of assisting cell growth, cell attachment and specific differentiation. Previous study has shown that the stiffness and chemical composition of the scaffolds mainly affect the proliferation and differentiation capacity of stem cells [51]. Nanomaterials-based 3D scaffolds with different stiffness and chemical composition provide an ideal platform for cell–cell/nanomaterials communications and the properties of scaffolds can be varied to promote differentiation of stem cells into specific lineages [16]. 3D nano-scaffold systems have proven to enhance osteogenic, neural, chondrogenic and odontogenic differentiation [89, 132–134].

### Application of nanomaterials in the differentiation of stem cells into specific lineages

The application of various nanomaterials in stem cell differentiation has been mentioned above, and a summary is listed in Table 1. Combination of nanomaterials and stem cells brings us powerful tools to generate various specific lineages like osteoblast, neural cell, cardiocytes, chondrocyte, hepatocyte-like cells, and so on. It is noteworthy each nanomaterial is so versatile that can be fabricated for many purposes to differentiate different stem cells (MSCs, ESCs, ADSCs, NSCs, iPSCs, ect.) into different lineages. For example, silica nanoparticles have no positive effect on the differentiation of stem cells, but they can be designed as nanocarrier to carry insulin to rMSCs for adipogenic differentiation [94], or delivery AA to hESCs for cardiac differentiation [15]. Similarly, aligned SWCNTs have been reported to promote osteogenic differentiation of hMSCs [131]. In addition, carboxylated MWCNTs can direct neural differentiation of hBMSCs [86], and poly(ε-caprolactone)-functioned SWCNT scaffolds can enhance cardiac differentiation of rMSCs [90]. As shown in Table 1, AuNPs, AgNPs, silica nanoparticles and polymeric nanoparticles are more suitable as additives or as carriers for stem cell differentiation. However, TiO_2_, GR, GO and CNTs as 2D/3D nano-scaffolds for stem cell differentiation are more worthy of study and exploration.

**Table 1:** A summary of the applications of nanomaterials in stem cell differentiation

Nanomaterials	Chemical modifications/components	Cell lineages generated	Cell sources	References
*Nanoparticles and nano-carriers*				
AuNPs		Osteogenic differentiation	mMSCs/hADSCs/hMSCs	[34, 44, 47]
AuNPs	Chitosan	Osteogenic differentiation	hADSCs	[49]
AgNPs		Osteogenic differentiation	mMSCs	[29]
AgNPs		Osteogenic differentiation	hUSCs	[57]
AgNPs	miR-148b	Osteogenic differentiation	hADSCs	[58]
GO		Dopamine neurons	mESCs	[33]
GQD		Osteoblasts and adipocytes	rBMSCs	[79]
Silica nanoparticles	Insulin	Adipogenic differentiation	rMSCs	[94]
Silica nanoparticles	AA	Cardiac differentiation	hESCs	[15]
Mesoporous silica nanoparticles	HNF3β plasmid DNA	Hepatocyte-like cells	miPSCs	[95]
Silica nanoparticles	PEDF siRNA	Self-renewal and differentiation	hCSCs	[96]
IONPs		Osteogenic differentiation	hBMSCs	[65]
IONPs	HSA/FGF2	Neuronal, adipogenic and osteogenic lineages	hMSCs	[67]
Barium titanate nanoparticles		Proliferation and differentiation	rMSCs	[68]
DNA nanotubes	Peptide RGDS	Neurons	mNSCs	[114]
Chitosan-based-microRNA nanoparticles	AntimiR-138	Osteogenic differentiation	rMSCs	[104]
Polymeric nanoparticles	RA	Neuronal differentiation	mNSCs/hiPSCs/mouse SVZ stem cells	[99, 100, 130]
Chitosan nanoparticles	Hepatocyte growth factor	Hepatocytes	mBMSCs	[129]
Polyethyleneimine complex nanoparticles	RA	Neuronal differentiation	mESCs	[98]
Polymeric nanoparticles	siSOX9 and RA	Neurons	mNSCs	[140]
*2D and 3D nano-scaffolds*				
AuNPs-loaded functionalized nanofibers	PCL/SF/AV/VitB12/GNP fibers	Cardiac differentiation	hMSCs	[51]
AuNPs-loaded hybrid nanofibers	BSA/PVA scaffolds	Cardiac differentiation	hMSCs	[46]
TiO_2_ nanotubes		Osteogenic differentiation	hADSCs/rBMSCs	[35, 62]
TiO_2_-coated CoCrMo		Osteogenic differentiation	hMSCs	[141]
TiO_2_ scaffolds		Osteogenic differentiation	hADSCs	[14]
GR/TiO_2_ heterojunction		Neurons	hNSCs	[142]
GR	Laminin-coated	Neurons	hNSCs	[76]
GR		Osteogenic differentiation	hMSCs	[41]
GO-PLGA nanofiber scaffolds		Osteogenic differentiation	hMSCs	[78]
rGO-collagen hybrid scaffold		Neural cells	rBMSCs	[77]
Graphene nanogrids		Osteogenic differentiation	hMSCs	[143]
Aligned SWCNTs		Osteogenic differentiation	hMSCs	[131]
SWCNTs		Adipogenesis	rMSCs	[87]
MWCNTs	Poly(ε-caprolactone)	Cardiac differentiation	hMSCs	[90]
MWCNTs	Carboxylated	Neural cells	hBMSCs	[86]
MWCNTs	PEG	Osteogenic differentiation	hMSCs	[40]
MWCNTs-incorporated nanocomposite scaffolds		Cartilage regeneration	hBMSCs	[88, 89]
Xanthan and magnetite nanoparticles hybrid scaffolds		Neurons	mESCs	[144]
PLLA/PBLG/collagen nanofibrous		Osteogenic lineages	Rabbits-ADSCs	[145]

Anyhow, in view of the inherent properties, ligands and the drug-loaded on the surface of nanomaterials have great influence on the differentiation, more research and efforts are needed to design suitable conditions for stem cell specific differentiation according to the characteristics of various nanomaterials.

## Conclusions

The rapid development of nanotechnology provides a variety of nanomaterials, especially metal nanoparticles, carbon nanomaterials, semiconductor nanomaterials, polymeric nanoparticles and DNA nanostructures, which are promising in regulating stem cell behavior and tissue regeneration [135, 136]. Nanomaterials can potently modulate the drug-loaded release or microenvironments involved in stem cell differentiation, and enhance their efficiency and safety [123]. The combination of stem cell research and nanomaterials offers new insights to treat various diseases, including cardiovascular disease, neurodegenerative diseases, bone tissue formation and regeneration [33, 129, 137, 138].

Among these nanomaterials, AuNPs are good conductors that can be used for deliver electrical stimulation on the differentiation of stem cells. Some other nanoparticles, such as AgNPs, silica nanoparticles, PEI, chitosan and DNA nanostructures, seem to have more advantages in drug delivery. However, due to most of the nanomaterials mentioned above are not biodegradable, chronic toxicity and other side effects should be noted when nanoparticles are used as supplements or nanocarriers for stem cell differentiation. Therefore, two kinds of biodegradable nanomaterials, chitosan and DNA nanostructures, are worth exploring as a nanocarrier for drug delivery. Moreover, TiO_2_, GO, GR and CNTs are ideal biocompatible and mechanically platforms that are worth exploring for 2D matrix supports or 3D nano-scaffolds to facilitate stem cell differentiation. Moreover, some nanomaterials exhibit concentration-dependent toxicity, which should be taken into account during the application of nanomaterials [52, 139].

As mentioned above, the application of nanomaterials in the modulation of stem cell differentiation mainly through three ways (nanoparticle suspension, 2D culture, 3D culture), and the functions and mechanisms of nanomaterials in the differentiation of stem cells are different. In addition to the inherent ability to promote stem cell differentiation, nanomaterials with special desired lineages or drug loadings will modulate the specific properties for stem cell differentiation, and its stiffness, alignment and several other parameters also proved to play an important role in stem cell fate. Because of its complexity, the exact mechanisms linking the nanomaterials and the fate of stem cells are not well studied. Most of the literatures have not been deeply studied on the mechanism of the differentiation of stem cells promoted by nanomaterials. Further researches are hence needed to elucidate the mechanisms and biological effects of nanomaterials on stem cell differentiation. In addition, in order to improve the cell response for specific differentiation, novel nanomaterials with appropriate nanobio-interface, specific physical, biochemical, and biomechanical cues also should be further developed.

Based on the main findings from above mentioned studies, it is reasonable to believe that the differentiation of stem cells modulated by nanomaterials has broad application prospects. Although the combined use of stem cells and nanomaterials in stem cell differentiation currently is still in preliminary research phase and far from being applied clinically, and there are still many challenges to be solved in the use of nanomaterials for stem cell differentiation, this strategy is still promising for the application of stem cell differentiation in stem cell therapy and will certainly have a breakthrough in the recent future.

## Abbreviations

SSCs: somatic stem cells
iPSCs: induced pluripotent stem cells
MSCs: mesenchymal stem cells
ADSCs: adipose-derived stem cells
NSCs: neural stem cells
BMSCs: bone-derived mesenchymal stem cells
USCs: urine-derived stem cells
TiO_2_: titanium dioxide nanoparticles
AuNPs: gold nanoparticles
AgNPs: silver nanoparticles
GR: graphene
GO: graphene oxide
IONPs: iron oxide nanoparticles
GQD: graphene quantum dots
CNTs: carbon nanotubes
MAPK: mitogen-activated protein kinase
HSCs: hematopoietic stem cells
BMP2: bone morphogenetic protein 2
RGDS: Arg-Gly-Asp-Ser
2D: two-dimensional
3D: three-dimension
HAS: human serum albumin
FGF2: fibroblast growth factor 2
PLGA: poly(lactic-co-glycolic acid)
PEG: polyethylene glycol
HNF3β: hepatocyte nuclear factor 3β
PEDF: epithelium-derived factor
RA: retinoic acid
SVZ: subventricular zone
HIF-1α: hypoxia inducible factor-1α
AA: ascorbic acid
